# Pavor Nocturnus in Children

**Published:** 1910-09-17

**Authors:** J. Allen


### THE GENERAL PRACTITIONER'S COLUMN.

[Contributions to this Column are invited, and, if accepted, will be paid for.]

### PAVOR NOCTURNUS IN CHILDREN.

### SOME POINTS IN TREATMENT.

By J. ALLEN.

Pavor nocturnus is one of the minor affections met with in children which, though not dangerous to life, is liable to entail much suffering on the part of the child and to cause much anxiety to parents. The treatment, while generally satisfactory, may occasionally baffle the resources of the medical attendant.

Many conditions seem to act as causative factors, among which may be mentioned enlarged tonsils and adenoids, phimosis, errors of refraction, worms, dyspepsia, and constipation. In a fair percentage of cases there is a rheumatic diathesis, while in a number a neurotic family history is found. Some are classed as idiopathic, while a few appear to be associated with more grave nervous affections, such, for instance, as epilepsy.

In dealing with pavor nocturnus it is necessary to consider the treatment during an attack, and the means to be adopted to prevent recurrence. During an attack every effort should be made to calm the child by gentle persuasion. The child should not be whipped or punished. A warm foot-bath and cold douches to the head may assist in allaying the state of nervous terror. If necessary, the mother or nurse should sit by the bedside until the child falls asleep again. If there is any suspicion of indigestion, some recommend that an emetic should be given, so as to rid the stomach of its unwelcome load. If persuasion fails to pacify the child, a hypnotic such as paraldehyde, bromural, potassium bromide, etc., may be given.

Of much greater importance is the question of the management of a child who is subject to attacks of night terror. In the first place a very careful examination should be made in order to find out if there is a possible causative factor, and if any such be discovered it should at once be remedied. For instance, enlarged tonsils and adenoids should be removed, errors of refraction corrected, genital irritation treated, and so on.

The diet should be carefully regulated. Plain and easily digested articles of food are the best. All sweets and cakes should be forbidden, and the carbohydrate intake should be strictly limited. Tea, coffee, and alcoholic beverages should not be allowed. Meals should be taken regularly, and if the child has been in the habit of eating between meals this bad practice must be stopped. Care should be taken to see that the evening meal is not a heavy one. Eustace Smith advises that an alkali should be given two or three hours after food.

The education of the child must be very carefully supervised. It is a great mistake to drive these children too hard at school, and it is a debatable point whether they should be punished for failing to have adequately prepared their daily task. Some of these children, far from being backward, are intellectually brilliant, and they rather require to be curbed in their too strenuous efforts after knowledge. These children should not be allowed to enter for competitive examinations, as the strain and excitement accruing to such cannot fail adversely to affect their unstable nervous systems. If a child is having attacks of night terror very frequently it may be advisable to order six months' complete rest from school work.

The child's amusements should also be carefully regulated. The healthy influence of moderate exercise is undoubted, but any games that tend to cause excitement should be discouraged.

General hygienic measures should be enforced. The sleeping apartment should be well ventilated and the window should be kept open night and day. The child should be encouraged to be in the open air as much as possible. Care should be taken not to overclothe the child, and at night time especially not to cover him with heavy bedclothes, as sweating may thus be produced, and it has been suggested that this may actually cause night terrors. Cold sponging (tepid at first if necessary) is invaluable and is most invigorating. For some sea-bathing is excellent, but it should only be permitted if followed by a healthy reaction. A child should not be forced to bathe in the sea if he appears disinclined to do so. A change of residence to the country or seaside is often advantageous.

The beneficial influence in the home of some capable person can hardly be over-estimated. It is most unfortunate if the child is left to the care of a neurotic mother or nurse. It would be much better to send him away with some friend who would be a congenial companion to him. It requires a person of firmness and tact and of a sympathetic nature successfully to undertake the charge of a child subject to night terrors. It is almost superfluous to mention that the child should not be told about ghosts, and story-books of too exciting a nature should, for the time being at any rate, be vetoed.

With regard to drugs, these are of less importance than the points that have just been considered. However, if the child regularly suffers from night terrors it is advisable to prescribe antipyrine, bromural, or some similar drug at bedtime. Leonard Guthrie advises that the "three bromides" should be administered in neurotic cases, and he has found that this combination can be continued without harm for a considerable period if moderate doses are given. In rheumatic cases salicylate of quinine will be found useful. In anæmic children iron or iron and arsenic should be ordered, while for debilitated subjects cod-liver oil can be employed with benefit.

Prognosis is on the whole good, and with careful treatment the condition can generally be cured. As the child becomes older there is less liability to suffer from pavor nocturnus. If the night terrors are associated with some other nervous affection, such as epilepsy, the prognosis will, of course, depend on that. Pavor nocturnus probably does not occur in the healthy child, and, in conclusion, it should be borne in mind that the condition is not one that should be neglected. As Dr. Still very aptly points out, the affection is the "slacken speed" to the engine-driver, which must never pass unheeded. Therefore thoroughly investigate every case and promptly treat the condition. Recollect also that prevention is better than cure, and in the case of neurotic children much can be done by proper care and training.

